# Barley Products of Different Fiber Composition Selectively Change Microbiota Composition in Rats

**DOI:** 10.1002/mnfr.201701023

**Published:** 2018-08-12

**Authors:** Cristina Teixeira, Olena Prykhodko, Marie Alminger, Frida Fåk Hållenius, Margareta Nyman

**Affiliations:** ^1^ Food for Health Science Centre Kemicentrum Lund University SE‐221 00 Lund Sweden; ^2^ Food and Nutrition Science Biology and Biological Engineering Chalmers University of Technology SE‐412 96 Göteborg Sweden; ^3^ Food Technology Engineering and Nutrition Kemicentrum Lund University SE‐221 00 Lund Sweden

**Keywords:** β‐glucan, arabinoxylan, barley malt, BSG, microbiota

## Abstract

**Scope:**

Several dietary fiber properties are suggested to be important for the profiling of the microbiota composition, but those characteristics are rather unclear. Whether different physico‐chemical properties of barley dietary fiber influence the gut microbiota composition is investigated.

**Methods and results:**

Seven diets containing equal amounts of dietary fiber from barley malts, brewer's spent grain (BSG), and barley extracts, resulting in varying amounts of β‐glucan, soluble arabinoxylan, and insoluble arabinoxylan in the diets were given to conventional rats. Malts increased microbiota alpha diversity more than BSG and the extracts. The intake of soluble arabinoxylan was related to *Akkermansia* and propionic acid formation in the cecum of rats, whereas β‐glucan and/or insoluble arabinoxylan were attributed to some potentially butyrate‐producing bacteria (e.g., *Lactobacillus*, *Blautia*, and *Allobaculum*).

**Conclusion:**

This study demonstrates that there is a potential to stimulate butyrate‐ and propionate‐producing bacteria in the cecum of rats with malt products of specific fiber properties. Moreover, BSG, a by product from beer production, added to malt can possibly be used to further modulate the microbiota composition, toward a higher butyric acid formation. A complex mixture of fiber as in the malts is of greater importance for microbiota diversity than purer fiber extracts.

## 1 Introduction

There is increasing evidence of a relation between the consumption of dietary fiber and its effects on colonic microbiota composition, and consequently to human health.

Dietary fibers are indigestible food components reaching the colon with the potential to be utilized by some species of the microbiota and promote the growth of others, for example, through pH changes and bacterial cross‐feeding mechanisms.^1^, ^2^ Upon microbiota fermentation, dietary fibers can yield different amounts and patterns of SCFA, mostly acetic‐, propionic‐, and butyric acids, which are rapidly absorbed by the colonocytes into the circulation, serving as energy source and precursors in anti‐inflammatory mechanisms.^3^

The characteristics of the dietary fiber components reaching the colon may affect the microbiota composition differently, but this is not yet well understood. Some studies suggest that an increase of *Lactobacillus* in human feces^4^ and in rat cecum^5^ after consumption of β‐glucan are due to the oligomeric form of β‐glucan rather than the polymeric form. According to in vitro studies, some *Bifidobacterium* and *Lactobacillius* strains can utilize xylan oligosaccharides but not β‐glucan.^6^ Furthermore, soluble arabinoxylan from wheat are considered to be butyrogenic^7^ and propiogenic,^8^ in humans and rats, respectively, and related with the increase of *Roseburia, Prevotella*, and *Bifidobacterium* in studies on rodents.^9^, ^10^

Barley is one of the most well‐adapted cereal crops to different environmental conditions. There is growing interest for its use in human nutrition, due to its high content of dietary fiber and associated positive health effects, such as reduced risk of cardiovascular diseases, type 2 diabetes, and colorectal cancer.^11^ β‐glucan is especially highlighted in this respect, but also arabinoxylan may be of interest, with physico‐chemical properties dependent on variety and processing conditions, and in this way being a potential gut microbiota modulator.^12^ Whole‐grain barley and especially barley malt resulted in higher levels of *Blautia* (butyrate‐producer) in the hindgut of rats compared to a fiber‐free control diet, and also of *Akkermansia*,^12^ a bacterium that has been related to reduced mucosa inflammation.^13^ A considerable increase of butyric acid formation was found in rats fed with malt compared with whole‐grain barley.^14^ This was suggested to be related to the changed characteristics of the β‐glucan due to increased β‐glucanase activity during malting,^15^ also causing the increased number of *Roseburia*, *Coprococcus*, and *Lactobacillus*.^12^ However, a change of other components during this process such as arabinoxylan cannot be excluded. Another interesting barley product is brewer's spent grain (BSG), a waste product from brewing industry. BSG contains high amounts of hemicelluloses and protein, depending on the barley variety and processing conditions used. BSG was suggested to have hypocholesterolemic effects,^16^ influence lipid metabolism,^17^ and to potentially act as a growth stimulator of *Bifidobacterium* and *Lactobacillus* in the distal colon.^18^

The aim of this study was to investigate whether barley malts and BSG with different fiber characteristics (β‐glucan, soluble and insoluble arabinoxylan), could affect the microbiota composition, the potential for SCFA formation, especially butyric‐ and propionic acid, and metabolic functions. For this purpose, conventional rats were fed diets containing three barley malts from different varieties or produced at different malting conditions, a barley BSG, a mixture of malt and BSG, or a fiber‐free control. Since arabinoxylan and β‐glucan are two of the main dietary fibers of interest in barley, groups fed diets containing extracts from barley especially rich in these polymers were also included.

## 2 Experimental Section

### 2.1 Test Materials

Five barley malt products and two barley extracts were selected and compared in the study due to their differences in contents of arabinoxylan and β‐glucan (**Table** 1): tipple malt (TM), cinnamon malt (CM), standard malt (SM), BSG, a mixture of TM and BSG (mixture), β‐glucan rich extract (BG extract), and arabinoxylan rich extract (AX extract).

**Table 1:** Contribution of dietary fiber components from the barley products in the diets, g per 100 g dry weight^a^

	Tipple malt	Cinnamon malt	Standard malt	BSG	Mixture^b^	AX extract	BG extract
Total Fiber	8 (20)	8 (33)	8 (10)	8 (2.8)	8 (10)	8 (87)	8 (49)
Arabinoxylan	2.6 (5.0)	2.3 (10)	3.2 (4.6)	3.0 (1.0)	2.8 (2.6)	5.7 (76)	2.2 (2.9)
β‐glucan	1.3	1.4	0.3	0.1	0.6	0.1	4.8
β‐glucan Mw (g mol^–1^) ×10^6^	1.4	1.3	1.0	0.4	1.1	0.07	1.6

a Values in parenthesis refer to the soluble proportion of the fiber (%)

b Composed of Tipple malt and BSG (70:30).

TM and CM were produced in a pilot plant at Lahden Polttimo Oy (Lathi, Finland), under nontraditional steeping conditions to preserve β‐glucan content (35 °C and 0.4% lactic acid) and kilned at 55−70−82 °C for 10−8−8 h, respectively. SM was steeped at 14.5 °C without lactic acid, and kilned at 55−86 °C for 20 h. BSG was a by‐product from the SM milling, mashed at 20−70 °C, and dried at 48−55 °C for a total of 20 h. Both SM and BSG were provided by an affiliated company to Lahden Polttimo Oy (Viking Malt AB, Halmstad, Sweden). To obtain a diet with intermediary content of β‐glucan and arabinoxylan in relation to the malt diets, a Mixture containing 70% TM and 30% BSG was also included. AX extract was purchased from Xylophane AB (Göteborg, Sweden) and BG extract from Lyckeby Starch AB (Kristianstad, Sweden), and used as reference samples, due to their high amounts of arabinoxylan and β‐glucan, respectively. AX extract was isolated from barley husk involving extraction at elevated pH, and according to the provider, resulting in AX of high molecular weight (Mw; ≈70 kDa). BG extract was obtained by mechanical fractionation of barley kernels, mostly originating from the aleurone layer cell walls and according to the provider, resulting in a β‐glucan with high Mw (1.6 × 10^6^). The malt products were milled to a particle size less than 0.5 mm for characterization analysis and incorporated into test diets. The extracts were used as provided in fine powder.

### 2.2 Diets

The design of the study resulted in eight diets (seven test diets and one control), composed of 12% casein, 1.2% dl‐methionine, and 2% choline chloride (Sigma–Aldrich, St. Lois, MO, USA), 5% maize oil (Ica, Solna, Sweden), 10% sucrose (Dan Sukker, Malmö, Sweden), and 0.8% vitamin and 4.8% mineral mixtures (Lantmännen, Malmö, Sweden) (Table S1, Supporting Information A). Furthermore, the seven test diets contained an equal amount of dietary fiber (80 g kg^−1^ dry weight) resulting in an amount of added barley product between 96 and 527 g kg^−1^. Wheat starch was added to adjust for the dry matter content and since it is completely digested, no fiber is delivered to the colon and there is no contribution of SCFA from this source.^19^ The control diet contained wheat starch as substitute to the fiber source (fiber‐free diet). The diets were prepared in house by mixing all the ingredients thoroughly in a mixer for 60 min, and given in the form of powder.

### 2.3 Characterization of Test Materials

Soluble and insoluble dietary fiber contents were measured according to an enzymatic gravimetric method.^20^ The neutral sugars of the isolated dietary fiber residues and fecal material were analyzed using a gas‐chromatographic method,^21^ and arabinoxylan content was estimated as the sum of arabinose and xylose in the fiber residues. The degree of fermentation was calculated as in Equation (1), during the five days of the experimental period.

(1) 1− grams  of  neutral  sugars  in  faeces / grams  of  neutral  sugars  consumed ×100

The content of β‐glucan was assessed with the mixed‐linkage β‐glucan assay kit (K‐BGLU, Megazyme International, Ireland). The average β‐glucan Mw of extractable β‐glucans was estimated with high‐performance size exclusion chromatography and fluorescence detection (HPSEC–FD) with Calcofluor post‐column complexation.^22^ Resistant starch was estimated by the difference between total and available starch, obtained by an enzymatic assay with KOH and/or digestion with α‐amylase and amyloglucosidase.^23^, ^24^ The amylose content was quantified with an amylose/amylopectin kit (Megazyme International, Ireland). The crude protein was quantified with an elemental analyzer (Flash EA 1112, Thermo Fisher Scientific Inc., Waltham, MA, USA). All analyses were made at least in duplicate.

### 2.4 Animal Study

Male Wistar rats (*n* = 56) with weights between 70 and 98 g were randomly divided into eight groups and caged individually. The experiment lasted for 12 days, during which rats were fed the test and control diets and the temperature and light were kept constant: 21 °C and 12 h light cycle. The food was restricted to 12 g dry weight per day, and water was given ad libitum similar to previous studies.^25^ The design of the experiment has been used in previous experiments, and has been shown to be enough to study changes in total fermentation of dietary fiber and SCFA formation (e.g., refs. [26, 27, 28]).

During the last 5 days of the experimental period, feces were collected daily, freeze‐dried, and milled before analysis of dietary fiber. There were no feed residues during any of the experimental days. After the experimental period, the rats were anesthetized by cutaneous injection with a mixture of Hypnorm, Dormicum, and sterile water (1:1:2) at a dose of 0.15 ml per 100 g body weight. The weights of cecal content and cecal tissue were noted. A portion of the cecum content (apex) was stored at  −80 °C for microbiota analysis, while the pH of the remaining cecal content was measured and stored at –40 °C until analysis of SCFA.

### 2.5 Cecum Analyses

SCFA in the caecum content were analyzed by GC after the material was homogenized (Ultra Turrax T25 basic, IKA WERKE) and centrifuged with 0.25 m HCl.^29^

The cecum apex was used for extraction of DNA and sequencing of V1–V3 region of 16S rRNA gene, which was performed by GATC Biotech (Konstanz, Germany; http://www.gatc-biotech.com) by using amplicon‐based method. Forward and reverse sequence primers for amplification were 5′AGAGTTTGATCCTGGCTCAG3′ and 5′ATTACCGCGGCTGCTGG3′, respectively. Genome Sequencer Illumina HiSeq accredited method was performed using HiSeq Rapid Run 300 bp paired‐end kit (Illumina, San Diego, California, USA). Raw sequencing data for both forward and reverse reads were received in a FASTQ format, accompanied by a raw‐data quality report for every sample. Since the amplicons were sequenced in both directions, using Next‐generation Sequencing Platforms that is only able to generate relatively short read lengths (<500 bp), the read pairs were merged to increase the overall read length by using FLASH v1.2.11 software tool (The Center for Computational Biology, Johns Hopkins University) with maximum mismatch density of 0.25. Next, combined pairs of every sample (89 ± 1%) with mean length of 524 bp were processed for quality filtering and accuracy of operational taxonomic units (OTU) assignment by open‐source bioinformatics pipeline, Quantitative Insights into Microbial Ecology (QIIME v 1.9.1). A total number of 27 858 485 reads were generated after quality filtering for 56 samples with a mean of 497 472.946 reads per sample (minimum 212 070 and maximum 840 486). The sequences were grouped into OTUs at a minimum of 97% similarity. Taxonomy was assigned using Greengenes database (v.13.8). In total, 431 observations were obtained after removing singletons and low‐abundance OTUs (<0.0001) resulted in seven OTUs ID at phylum and 38 OTUs ID at genus levels. Next, alpha and beta diversity was analyzed at the even depth of 200 000 sequences per sample, retaining all samples in the analysis. It is worth mentioning that the number of observations before filtering and cutoff steps was 3235, giving zero approaching values for most of the taxonomic genera. The raw data and biom summary tables with sample reads and at genus level for each group before and after filtering are available in the Supporting Information (B for raw data and C for biom summary tables). Additionally, the QIIME statistics, including test for alpha‐diversity comparison and groups’ comparisons OTU frequencies (Kruskal–Wallis with Bonferroni correction) are also available in the Supporting Information D).

### 2.6 Prediction of Bacterial Metagenomes Using Phylogenetic Investigation of Communities by Reconstruction of Unobserved States

Potential metabolic functions from the gut bacteria were analyzed by inferring metabolic capacity from the 16S rRNA gene sequencing data using an open‐source software, Phylogenetic Investigation of Communities by Reconstruction of Unobserved States (PICRUSt).^37^ Results were thereafter analyzed for statistical significance using LEfSE with LDA score cutoffs of 2.0 (Figure S2a and S2b, Supporting Information A) and 3.0 (Figure 5).

### 2.7 Statistical Analyses

One‐way analysis of variance and Tukey's post‐hoc tests were used to evaluate the difference between the treatments, and post‐hoc Games–Howell test was used for nonhomogeneous data, with significance at *p* < 0.05. Correlations were evaluated with two‐tailed Pearson's tests: weak (±) 0.25–0.50; moderate (±) 0.50–0.75; strong (±) 0.75–1. Statistical analysis was performed with SPSS Statistics. All weights are on dry weight basis, except for cecal content, cecal tissue, and SCFA.

## 3 Results and Discussion

### 3.1 Dietary Fiber

To investigate the possibility to modulate microbiota composition and metabolic effects with barley malt products, the diets in the present study were designed to have the same quantity of total fiber (80 g kg^−1^; Table S1, Supporting Information A), but with different fiber characteristics of β‐glucan and arabinoxylan (Table 1).

Malts from the cultivar Tipple (TM), and Cinnamon (CM) were selected due to their comparatively high β‐glucan content (mean 1.4 ± 0.1 g per 100 g) and Mw (mean 1.3 ± 0.1 × 106 g mol^−1^), and these products also contributed with higher proportions of soluble fiber in the diets (20–33%) than SM (0.3 g per 100 g β‐glucan content, 1 × 106 g mol^−1^ Mw, and 10% soluble fiber). The reason to these differences is that SM is processed at traditional malting conditions to provide highly degraded β‐glucan (Table 1). Another dissimilarity between the malts was that the CM diet contributed with higher proportions of soluble arabinoxylan (10%) than the TM and SM diets (mean 4.8 ± 0.2%). Contents of protein, resistant starch, and amylose were very similar among the malt products (Table S2, Supporting Information A).

BSG was obtained as a by‐product from the mashing step of the malting process, where most of the soluble compounds of the malt are removed. As a result, the product had lower contents of soluble fiber, soluble arabinoxylan, β‐glucan, and β‐glucan Mw than the malts, but the contribution of total arabinoxylan to the diet was similar as with the SM diet (3.1 ± 0.1 g per 100 g). The diet composed of a mixture of TM and BSG (70:30) resulted in an intermediary content of β‐glucan and soluble arabinoxylan compared to the other malts and BSG diets and was used to evaluate the effect of adding BSG to malt.

To better understand the effect of arabinoxylan and β‐glucan on the gut microbiota composition, the effects of two barley‐based extracts rich in arabinoxylan (AX) or β‐glucan (BG) were studied. Diets containing AX and BG extracts had higher proportions of soluble fiber (87% and 49%, respectively) compared with the malts and BSG diets. The BG extract diet had the highest β‐glucan content (4.8 g per 100 g) of highest Mw (1.6 × 10^6^ g mol^−1^), and the AX extract contributed with the highest proportion of soluble arabinoxylan (76%).

### 3.2 Animal Experiment

The rats remained healthy and were active throughout the study. The daily feed intake was 12.0 ± 0.1 g dry weight, and the weight gain 11−13 g per rat during the last 5 days of the experiment (Table S3, Supporting Information A).

Cecal pH was higher for rats fed TM, AX, and BG extracts compared with BSG diet (mean 7.1 vs 6.3 with BSG). The cecal content was higher with AX and BG extracts than with the control (2.0 and 1.6 g, respectively vs 0.9 g for the control, *p* < 0.05). The fecal weight was higher in rats fed diets containing SM and BSG (6.3 and 5.7 g, respectively), than in rats fed the mixture, TM, and CM (4.6−5.0 g), AX extract (3.8 g), BG extract (2.9 g), and the fiber‐free control diet (1.5 g). This could be expected since both SM and BSG contained higher amounts of insoluble fiber, known be less fermented by the microbiota, mainly contributing to fecal bulk.

### 3.3 Fermentation of Dietary Fiber Polysaccharides

Total dietary fiber fermentation was higher in rats fed diets containing AX and BG extracts (86% and 85%, respectively), than in those rats fed CM, TM, and mixture diets (36−44%), and SM and BSG diets (21−23%) (Table S4, Supporting Information A), which corresponded with the higher content of soluble fiber and β‐glucan content, and/or Mw in the diets (Table 1). Details of the degree of fermentation for each neutral sugar are in Table S4, Supporting Information A.

### 3.4 SCFAs in Cecum of Rats

Acetic, propionic, and butyric acid were the major SCFAs found in the cecum of rats. As expected, the cecal content of total SCFA was lower in rats fed the fiber‐free control diet than in those fed AX and BG extracts (38 vs mean 78 μmol, *p* < 0.05) (Table 3). Acetic acid was higher in rats fed AX extract than those fed TM (53 vs 31 μmol, *p* < 0.05), and also the content of propionic acid compared with rats fed SM (14 vs 8 μmol, *p* < 0.05). Despite the different fiber composition in CM compared with BSG (CM had high proportions of β‐glucan and soluble arabinoxylan, whereas BSG had high proportions of insoluble arabinoxylan) both resulted in the highest butyric acid content, and it was significantly higher than in rats fed the control diet (7.3–6.7 vs 3.4 μmol, *p* < 0.05).

Taking the characteristics of the fiber in consideration, diets with more soluble arabinoxylan contributed to a higher proportion of propionic acid in the cecum of rats (Table 1 and 3), which was especially seen in rats fed AX extract. High proportions of propionic acid in the cecum of rats have also been related to the content of soluble arabinoxylans in a previous study.^8^ Interestingly, soluble fiber, soluble arabinoxylan, β‐glucan, and β‐glucan Mw were all positively correlated with the proportion of propionic acid, but negatively with acetic acid in the cecum of rats fed malt/BSG diets (**Table** 2). No correlations were found with the proportion of cecal butyric acid.

**Table 2:** Correlation of dietary fiber intake with cecal proportion of SCFA and the most abundant phylum levels in rats fed the malt and BSG diets (tipple malt, cinnamon malt, standard malt, standard BSG, and mixture)

	% Soluble fiber	Total arabinoxylan	% Soluble arabinoxylan	β‐glucan	β‐glucan Mw
% Acetic	−**	+**	−**	−**	−**
% Propionic	++***	−**	++***	++***	+**
% Butyric	NC	NC	NC	NC	NC
p *Actinobacteria*	NC	NC	NC	NC	NC
p *Bacteroidetes*	−**	+**	−*	−*	NC
p *Firmicutes*	+*	−**	NC	NC	NC

*, **, ***, denotes significance at the level of *p* < 0.1, *p* < 0.05, *p* ≤ 0.001, respectively (ANOVA); NC, no correlation

Plus/minus symbol indicates the positive/negative correlation between SCFA and bacteria at different correlation coefficient ranks, in absolute values: + or −, 0.25< r < 0.50; ++ or − −, 0.50 < r < 0.75; +++ or − − −, 0.75 < r < 1.00.

Due to practical reasons, to be able to finish the study for the groups (two to three groups per day), we started early in the morning, and consequently the test diets were removed from some of the rats more than 6 h before collection of the cecum content. During this time the SCFA might already have been absorbed and regressed to fasting levels, which may explain the comparatively lower values in the present study to studies with similar design.^12^, ^15^

### 3.5 Gut Microbiota Composition

#### 3.5.1 Diversity

**Figure 1:**
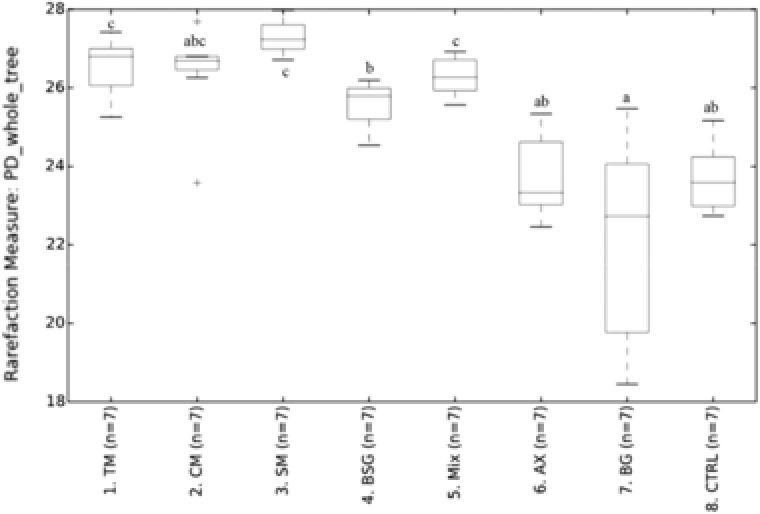
Alpha diversity. Alpha diversity indexes of PD (phylogenetic diversity) whole tree in rats fed test diets containing barley products or a control diet. Groups with different letters are significantly different. (TM, tipple malt; CM, cinnamon malt; SM, standard malt; BSG, brewers’ spent grain; Mix, mixture of TM and BSG 70:30; AX, arabinoxylan extract; BG, β‐glucan extract; CTRL, free‐fiber control).

A microbial community with low alpha diversity (within samples) has been linked to obesity, Crohn's disease, and ulcerative colitis.^30^, ^31^ Notably, diets containing TM, SM, and mixture generally resulted in higher cecal alpha diversity within microbial community than rats fed the control diet, or diets containing AX or BG extracts (**Figure** 1). This suggests that a more complex mixture of fibers as found in the malts promoted the growth of a greater number of species, and would thus be interesting in relation to some chronic diseases.

**Figure 2:**
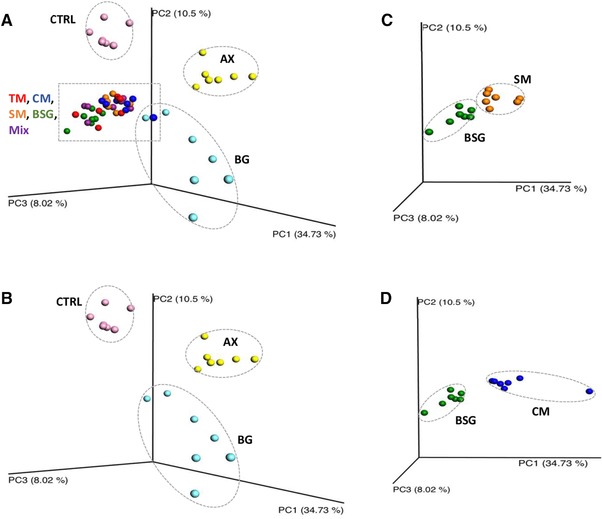
Beta diversity. Principal coordinate analysis of weighted Unifrac distance metrics based on the taxonomic similarities between samples at genus level. A) Groups fed test diets containing barley products or a fiber‐free control group. Barley malt/BSG groups are different from groups fed CTRL, BG, and AX. B) Groups fed CTRL, BG, and AX extract display different genus profile. C) BSG is different from SM, and (D) groups fed BSG are different from CM. (TM, tipple malt; CM, cinnamon malt; SM standard malt; BSG, brewers’ spent grain; Mix, mixture of TM and BSG 70:30; AX, arabinoxylan extract; BG, β‐glucan extract; CTRL,fiber‐free control).

A clear clustering of groups in the principal coordinate analysis (PCoA) of beta diversity (between samples from the same habitat) showed that rats fed malts/BSG diets had rather similar microbial communities, but was completely different from the gut microbiota in rats fed Control, AX, and BG extracts (**Figure** 2A), which also were markedly different from each other (Figure 2B). Furthermore, between the malt/BSG products, BSG displayed a distinct grouping compared with CM and SM (Figure 2C and 2D), which could be related to the differences in content of soluble arabinoxylan (Table 1).

#### 3.5.2 Relation between Specific Dietary Fiber Components and Microbiota Composition

**Figure 3:**
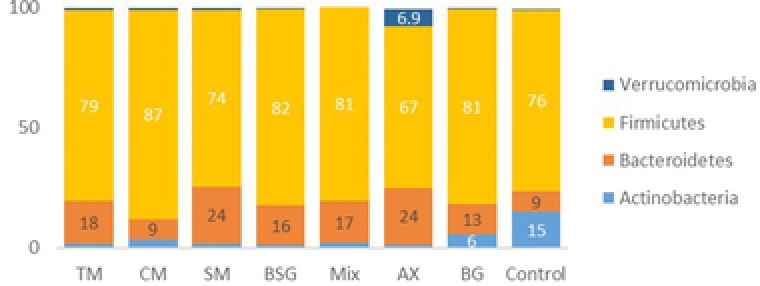
Relative abundance (%) at the phylum level. Relative abundance (%) of bacterial taxa at the phylum level in the caecum apex of rats fed test diets containing barley products or a control diet. (TM, tipple malt; CM, cinnamon malt; SM, standard malt; BSG, brewers’ spent grain; Mix, mixture of TM and BSG 70:30; AX, arabinoxylan extract; BG, β‐glucan extract; Control, free‐fiber control).

The most abundant phyla in cecum of all groups were Firmicutes (73−87%) and Bacteroidetes (9−24%) (**Figure** 3). Groups fed BG extract and the fiber‐free control had also rather high cecal amounts of Actinobacteria (6% and 15%, respectively), while considerably lower amounts of this phylum were found in caecum of rats fed barley malt products and AX extract (1−3.5%). Verrucomicrobia was exceptionally high in the cecum of rats fed AX extract compared with the other groups (6.9% vs ≤0.8%, *p* < 0.05).

The various diets thus appear to stimulate different bacterial species (at phylum level), in the cecum of rats. By relating the composition of the dietary fiber included in the diets with the abundance of bacteria, Bacteroidetes were negatively correlated with the intake of β‐glucan, proportion of soluble fiber, and soluble arabinoxylan (–0.314, –0.358, and –0.307, respectively) and positively correlated with total amounts of arabinoxylan in the diet (0.465) (Table 2). On the contrary, Firmicutes were positively correlated with soluble fiber (0.304) and negatively correlated with total arabinoxylan (–0.439). This may explain the differences of Bacteroidetes in cecum of rats fed the malted products CM and SM (9% vs 24%, respectively), where SM contained lower proportions of β‐glucan, soluble fiber, and soluble arabinoxylan than CM. Furthermore, the stimulation of Verrucomicrobia in cecum of rats fed AX extract compared with the other groups, could possibly be due to its high content of soluble arabinoxylan compared with the other barley products.

**Figure 4:**
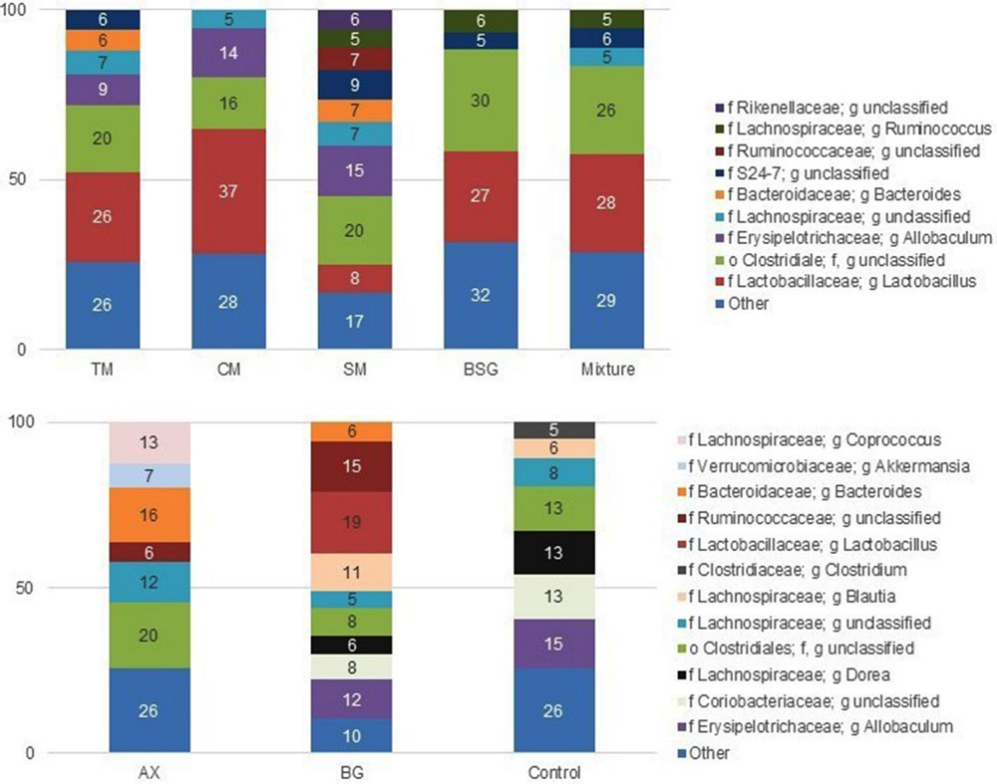
Relative abundance (%) at the genus level. Relative abundance (>5%) of bacterial taxa at the genus level in the caecum apex of rats fed test diets containing barley products or a control diet. TM, tipple malt; CM, cinnamon malt; SM, standard malt; BSG, brewers’ spent grain; Mix, mixture of TM and BSG 70:30; AX, arabinoxylan extract; BG, β‐glucan extract; Control, free‐fiber control.

**β‐glucan**: Rats fed BG extract had highest cecal amounts of the unclassified genera *Ruminococcaceae* (15% vs <7%, *p* < 0.05) (**Figure** 4). Furthermore, *Lactobacillus* was one of the most abundant genus in the cecum of rats fed malt/BSG diets and BG extract, and the amounts were positively correlated with β‐glucan content and Mw (Table S5, Supporting information A). Some *Lactobacillus* strains have been found to grow in the presence of oligosaccharides but not on polymeric β‐glucan.^5^, ^6^ It may therefore be questioned, whether some *Lactobacillus* in rats fed CM containing high β‐glucan Mw have used β‐glucans being depolymerized by other bacteria. Furthermore, β‐glucan in barley malt products might not be the only preferred substrate for *Lactobacillus*. Interestingly, the BSG diet had the lowest content of β‐glucan, but *Lactobacillus* was one of the most abundant genera in the cecum of rats fed this diet (27%). Also in other studies, barley has shown to increase the number of *Lactobacillus* in comparison with wheat, a cereal low in β‐glucan.^32^

By using the software tool LEfSe to compare bacterial taxa at species level, we found that CM increased the relative abundance of *Lactobacillus reuteri* (Figure S1, Supporting information A), a probiotic used for treatment and prevention of rotaviral diarrhea,^33^ and a suppressor of NF‐κB and TNF‐α inflammation markers.^34^, ^35^

*Blautia* was more abundant in rats fed BG extract, and these bacteria were previously found in the cecum of rodents fed whole‐grain diets^36^ and also in cecum of rats fed whole‐grain barley and barley malt.^12^ It is suggested that these bacteria benefit from the hydrogen as a product of glucan fermentation.^36^ Thus, they could have grown due to the depolymerization of β‐glucan from the BG extract diet.

**Arabinoxylan**: Total and soluble arabinoxylan affected the microbiota species differently. Unclassified *Rikenellaceae*, unclassified *S24‐7*, unclassified *Clostridiales*, *Ruminococcus*, *Odoribacter*, and *Oscillospira* are reported to be potentially butyrogenic^37^ and were more abundant in the cecum of rats fed diets high in total arabinoxylan but low in soluble arabinoxylan (TM, SM, BSG, and mixture) (Figure 4 and Table S6, Supporting information A), and negatively correlated with soluble fiber (Table S5, Supporting information A), suggesting that insoluble arabinoxylan is one of the preferred substrates for these taxa. On the other hand, soluble arabinoxylan but not total arabinoxylan favored the growth of *Clostridium*, *Blautia*, *Allobaculum*, *Coprobacillus*, although in rather low abundance (<5%), and of *Akkermansia* in cecum of rats fed malt/BSG. However, these bacterial species were also correlated with substrates containing high content of β‐glucan and β‐glucan Mw. Thus, the substrate preference between arabinoxylan and β‐glucan was not that clear. *Akkermansia* was one of the most abundant genus in the cecum of rats fed AX extract (7%, Figure 4), suggesting that its growth was more dependent on soluble arabinoxylan content than the other taxa. A decrease of *Akkermansia muciniphila*, the only known species of this genus, has been related to an increased risk to develop ulcerative colitis and obesity.^38^ Intake of water‐extractable arabinoxylan derived from wheat has previously been linked with an increased formation of propionic acid in the cecum of rats.^8^ Also in the present study, diets containing higher proportions of soluble arabinoxylan contributed with a higher cecal proportion of propionic acid (Table 2 and 3), which could especially be seen in cecum of rats fed AX extract, which could be the reason for the higher abundance of *Akkermansia* with this material.

**Table 3:** SCFAs in cecum of rats fed diets containing barley products (μmol)

	TM	CM	SM	BSG	Mixture	AX extract	BG extract	Control
Acetic	31^a^ ± 3	44^ab^ ± 6	38^ab^ ± 3	44^ab^ ± 4	41^ab^ ± 6	53^b^ ± 5	43^ab^ ± 7	25^a^ ± 2
Propionic	7^a^ ± 1	11^ab^ ± 2	8^a^ ± 1	9^ab^ ± 1	8^a^ ± 1	14^b^ ± 1	11^ab^ ± 2	8^a^ ± 1
Butyric	4.6^ab^ ± 0.7	7.3^b^ ± 0.9	5.3^ab^ ± 0.6	6.7^b^ ± 0.7	6^ab^ ± 0.6	6.1^ab^ ± 0.6	6.4^ab^ ± 1.2	3.4^a^ ± 0.3
Minor^a^	2.9^ab^ ± 0.4	3.7^ab^ ± 0.5	3.1^ab^ ± 0.3	3^ab^ ± 0.3	3^ab^ ± 0.4	4.5^b^ ± 0.5	4^ab^ ± 0.7	2.3^a^ ± 0.3
Total	45^ab^ ± 4	66^ab^ ± 9	55^ab^ ± 5	62^ab^ ± 5	59^ab^ ± 9	78^b^ ± 7	78^b^ ± 17	38^a^ ± 3

Values with different superscript letters (^a‐b^) in the same row are significantly different at *p* < 0.05

a Isobutyric, isovaleric, valeric, heptanoic, and caproic acid.

There was no direct correlation between *Prevotella*, *Coprococcus*, and *Dorea* and the fiber components in the malt/BSG diets. However, *Dorea* was more abundant in rats fed the BG extract and the control, while *Prevotella* and *Coprococcus* seemed to have a preference for AX extract but not for β‐glucan.

**Lignin‐like** substances and **resistant starch**: these were also part of the total fiber intake, especially in malt/BSG diets, but they were not correlated with any taxa.

#### 3.5.3 Comparison of Tipple Malt, BSG, and Mixture

Mixture diet (70% TM and 30% BSG) was also used to evaluate the possibility of adding BSG, a by‐product of low cost, to malt to modulate microbiota, which revealed to be a relatively easy approach for this purpose. Beta diversity and microbiota composition in rats fed TM, BSG and Mixture was similar at the phylum level (Figure 2A), but different at the genus level within *Firmicutes* (Table S6, Supporting information A). *Coprococcus*, a well‐known butyrate producer, was higher in rats fed mixture diet than in rats given the TM diet (1.2% vs 0.4%, *p* < 0.05), which might be associated with the comparatively higher butyric acid formation in cecum of rats fed BSG compared with TM. Furthermore, *Bacteroides* and *Allobaculum*, both reported to counteract adiposity and insulin resistance,^38^, ^39^ were more abundant in cecum of rats fed TM than the Mixture (Table S6, Supporting information A). In this study, there was no correlation between β‐glucan intake from malt and BSG diets and in the cecal number of *Bacteroides*, which contrasts with studies in feces of man.^4^

#### 3.5.4 Prediction of Functionality of Cecal Microbial Communities

**Figure 5:**
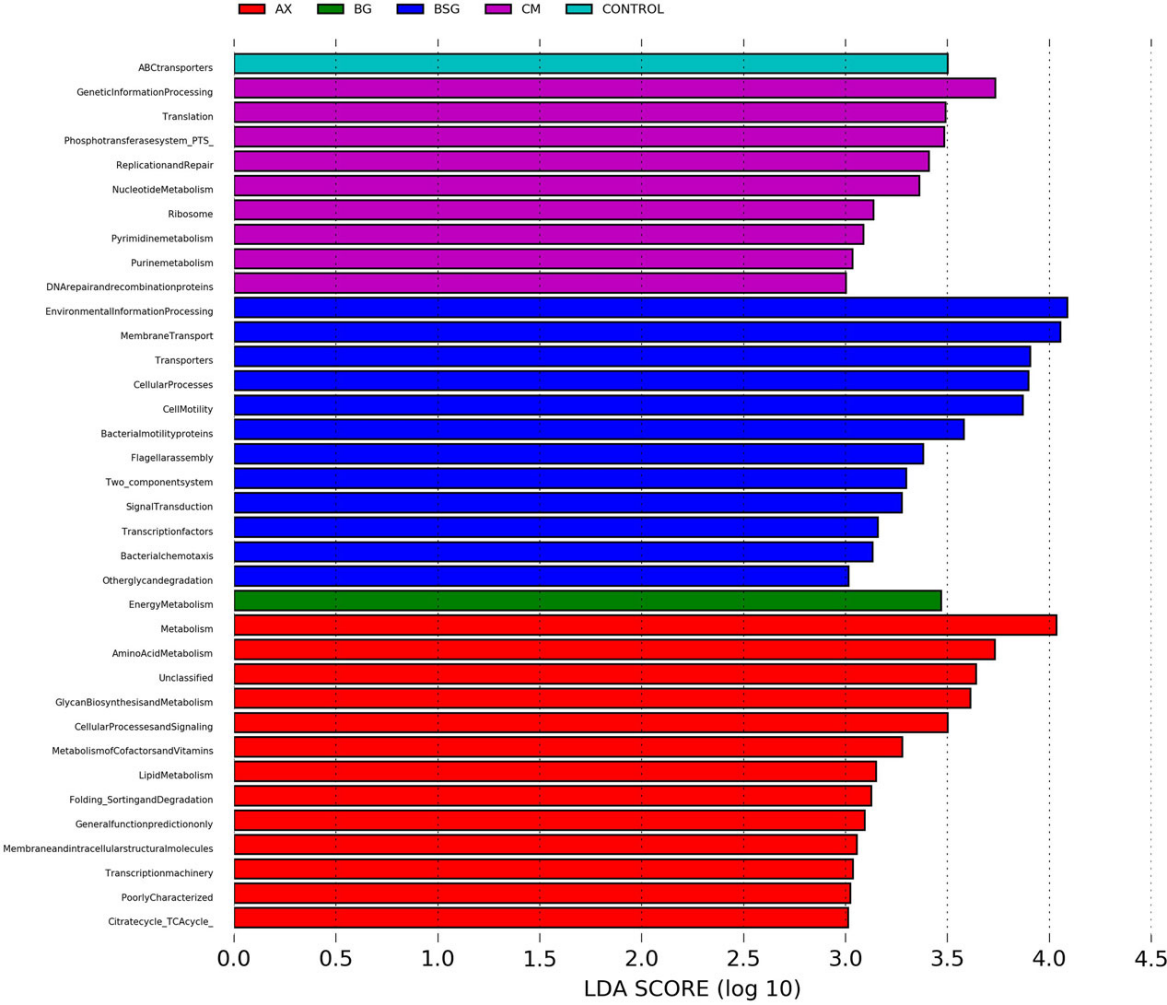
Prediction of bacterial metagenomes. PICRUSt analysis of 16S rRNA gene sequencing data. Functional microbial genes shown were enriched in the respective groups and had an LDA score higher than 3.

PICRUSt analysis together with LEfSe detected a total of 1, 9, 12, 1, and 13 genes enriched in the cecum of rats fed control, CM, BSG, BG extract, and AX extract, respectively (**Figure** 5). Two genes specialized in environmental information processing (both membrane transporters) were enriched in the control group and CM group. In the group fed BG extract, only one gene was enriched, specialized in energy metabolism. Genes enriched in CM were involved in genetic information processing (two genes in translation and two in replication and repair) and metabolism (two genes in membrane transport). With BSG, the genes found were mostly involved in environmental information (two genes in membrane transport and two in signal transducing) and cellular processes, all connected to cell motility (four genes). One of these cell motility genes was related to flagella assembly, which enables bacterial adhesion and invasion. Their relation with the colon is not straightforward, since flagella can be present in some harmful bacteria triggering inflammation, or help beneficial bacteria to adhere to the mucosa.^40^ Five genes enriched in AX extract were involved in diverse metabolisms (carbohydrate, lipid, amino acid, glycan, and co‐factors and vitamins). TCA (tricarboxylic acid) cycle present in the group fed AX extract generates energy derived from carbohydrates, which may explain a high degree of fermentation, especially of arabinoxylan. Furthermore, in the TCA cycle one of the precursors of butyric‐ and propionic acid (succinic acid) is produced, suggesting a high formation of these SCFA as indicated by slightly higher amounts detected in cecum of rats fed AX extract (Table 3). The genes related with glycan metabolism may reflect the high abundance of the mucus degrading bacteria *Akkermansia* in the cecum of rats fed AX extract.^41^

## 4 Conclusions

The stimulation of most bacterial species was related to the content of soluble fibers, including soluble arabinoxylan, β‐glucan and/or β‐glucan Mw and thus only a few with insoluble arabinoxylan. *Akkermansia* was more abundant in rats fed diets rich in soluble arabinoxylan and formed high cecal amounts of propionic acid, while the butyrogenic *Blautia*, *Allobaculum*, and *Lactobacillus* were more abundant in diets rich in β‐glucan. Of the malts, CM was particularly interesting as rats fed this product appear to give a high alpha diversity, stimulate the caecal abundance of *Allobaculum*, *Blautia*, and *Lactobacillus*, as well as butyric acid. Furthermore, BSG, with its distinct fiber composition, stimulated the cecal abundance of *Lactobacillus* and also butyric acid. This reinforces that different microbial communities with different substrate preferences can induce the formation of the same SCFAs, or that certain taxa, such as *Lactobacillus*, are little dependent of substrate preference. The addition of BSG to TM (i.e., mixture diet) resulted in an intermediary abundance of some taxa, and a slight increase in cecal butyric acid and butyrogenic bacteria, which shows the possibility of using BSG as a food ingredient to modulate the microbiota composition and function.

## Ethics Approval and Consent to Participate

The animal experiments were approved by the Ethics Committee for Animal Studies at Lund University (Ethical approval number M114‐15).

## Conflict of Interest

F.F.H. is shareholder in ProPrev AB. The other authors declare that they have no competing interests.

## Supporting information

Supporting Information

Supporting Information

Supporting Information

Supporting Information
