# Supplementary material for: Barley Products of Different Fiber Composition Selectively Change Microbiota Composition in Rats
Source: Mol Nutr Food Res. 2018 Aug 12;62(19):1701023. doi: 10.1002/mnfr.201701023 (PMC6175208; doi:10.1002/mnfr.201701023)
Supplement: Supplementary file 1 — Supporting Information [file MNFR-62-na-s001.docx]

**Supporting information A**

**Table S1.** Composition of test diets (g/kg), dry weight basis.

|  | Tipple malt | Cinnamon malt | Standard malt | BSG | Mixture ^1^ | AX extract | BG extract | Control |
| --- | --- | --- | --- | --- | --- | --- | --- | --- |
| Flour ^2^ | 424 | 359 | 527 | 148 | 271 | 96 | 147 | 0 |
| Wheat starch | 247 | 312 | 144 | 523 | 400 | 575 | 524 | 671 |
| Basal diet ^3^ | 329 | 329 | 329 | 329 | 329 | 329 | 329 | 329 |

^1^ Composed of Tipple malt and BSG (70:30).
^2^ Corresponding to 80 g dietary fibre per kg of diet.
^3^ Containing (g/kg):  364.5 casein, 3.6 DL-methionine, 151.8 maize oil, 6 choline chloride, 303.8 sucrose, 24.3 vitamin mixtures* and 145.8 mineral mixtures**

*Containing (g/kg): 0.62 menadione, 2.5 thiamin hydrochloride, 2.5 riboflavin, 1.25 pyridoxine hydrochloride, 6.25 calcium pantothenate, 6.25 nicotinic acid, 0.25 folic acid, 12.5 inositol, 1.25 p-aminobenzoic acid, 0.05 biotin, 0.00375 cyanocobalamin, 0.187 retinol palmitate, 0.00613 calciferol, 25 d-α-tocopheryl acetate, 941.25 maize starch (Lantmännen, Stockholm, Sweden)

**Containing (g/kg): 0.37 CuSO4·5H2O, 1.4 ZnSO4·7H2O, 332.1 KH2PO4, 171.8 NaH2PO4·2H2O, 324.4 CaCO3, 0.068 KI, 57.2 MgSO4, 7.7 FeSO4·7H2O, 3.4 MnSO4·H2O, 0.02 CoCl·6H2O, 101.7 NaCl, 0.019 chromium(III)chloride and 0.011 sodium selenite.

**Table S2.** Contribution of non-dietary fibre components from barley products in the diets, g/100 g dry weight.

|  | Tipple malt | Cinnamon malt | Standard malt | BSG | Mixture ^1^ | AX extract | BG extract | Control |
| --- | --- | --- | --- | --- | --- | --- | --- | --- |
| Resistant starch | 2.5 | <0.1 | 1.2 | 0.3 | 1.5 | 0.2 | 0.1 | 0 |
| Amylose | 7 | 0.6 | 9 | 1.9 | 6 | 0.3 | 0.2 | 0 |
| Protein | 3.4 | 3.3 | 4.4 | 2.5 | 2.9 | 0.7 | 1.2 | 0 |

^1^ Composed of Tipple malt and BSG (70:30).

**Table S3.** Body, caecal content and tissue, faecal weights (g) and caecal pH in rats fed diets containing barley products.

|  | Tipple malt | Cinnamon malt | Standard malt | BSG | Mixture | AX extract | BG extract | Tipple malt |
| --- | --- | --- | --- | --- | --- | --- | --- | --- |
| Body weight gain | 13^a^ ± 0.2 | 13^a^ ± 0.2 | 13^a^ ± 0.2 | 13^a^ ± 0.2 | 13^a^ ± 0.1 | 11^a^ ± 0.2 | 12^a^ ± 0.0 | 13^a^ ± 0.1 |
| Caecal pH | 7.0^bcd^ ± 0.1 | 6.4^ab^ ± 0.1 | 6.6^abc^ ± 0.1 | 6.3^a^ ± 0.1 | 6.6^abc^ ± 0.1 | 7.2^d^ ± 0.1 | 7.1^cd^ ± 0.2 | 6.6^abc^ ± 0.1 |
| Caecal content | 1.1^ab^ ± 0.1 | 1.4^abc^ ± 0.2 | 1.5^abc^ ± 0.2 | 1.3^ab^ ± 0.1 | 1.3^ab^ ± 0.1 | 2.0^c^ ± 0.1 | 1.6^bc^ ± 0.2 | 0.9^a^ ± 0.0 |
| Caecal tissue | 0.46^bc^ ± 0.02 | 0.51^cd^ ± 0.03 | 0.50^bcd^ ± 0.02 | 0.41^ab^ ± 0.01 | 0.42^abc^ ± 0.01 | 0.56^de^ ± 0.01 | 0.61^e^ ± 0.03 | 0.35^a^ ± 0.01 |
| Faecal dry weight | 4.8^d^ ± 0.1 | 4.6^d^ ± 0.2 | 6.3^e^ ± 0.1 | 5.7^e^ ± 0.1 | 5.0^d^ ± 0.1 | 3.8^c^ ± 0.1 | 2.9^b^ ± 0.2 | 1.5^a^ ± 0.1 |

Values with different letters (^a-e^) in the same row are significantly different at p<0.05.
* Significantly different at p<0.1

**Table S4.** Composition of (g/100g dietary fibre) and degree of fermentation (%) of dietary fibre in rats fed barley products.

|  | Tipple malt | Cinnamon malt | Standard malt | BSG | Mixture | AX extract | BG extract |
| --- | --- | --- | --- | --- | --- | --- | --- |
| *Composition* |  |  |  |  |  |  |  |
| Arabinose | 17 | 15 | 19 | 18 | 17 | 18 | 10 |
| Xylose | 35 | 31 | 41 | 42 | 39 | 71 | 17 |
| Mannose | 2 | 2 | 1 | 1 | 1.5 | 1 | 3 |
| Galactose | 5 | 2 | 4 | 2 | 3.5 | 2 | 2 |
| Glucose | 42 | 50 | 35 | 37 | 39 | 8 | 68 |
|  |  |  |  |  |  |  |  |
| *Degree of fermentation* |  |  |  |  |  |  |  |
| Arabinose | 53^ab^ ± 3 | 53^ab^ ± 2 | 48^ab^ ± 2 | 46^a^ ± 2 | 54^b^ ± 1 | 98^d^ ± 0 | 90^c^ ± 1 |
| Xylose | 37^a^ ± 4 | 37^a^ ± 2 | 30^a^ ± 2 | 32^a^ ± 1 | 39^a^ ± 2 | 98^c^ ± 0 | 81^b^ ± 1 |
| Mannose | 59^bc^ ± 2 | 70^c^ ± 1 | 34^a^ ± 5 | 38^a^ ± 5 | 56^b^ ± 3 | 25^a^ ± 3 | 88^d^ ± 1 |
| Galactose | 64^d^ ± 2 | 13^a^ ± 4 | 41^b^ ± 2 | 22^a^ ± 2 | 54^c^ ± 2 | 55^cd^ ± 1 | 51^c^ ± 2 |
| Glucose | 38^cd^ ± 3 | 50^d^ ± 3 | 9^a^ ± 4 | 12^ab^ ± 2 | 28^c^ ± 4 | 24^bc^ ± 4 | 89^e^ ± 1 |
| Total | 40^b^ ± 3 | 44^b^ ± 3 | 21^a^ ± 4 | 23^a^ ± 2 | 36^b^ ± 3 | 86^c^ ± 3 | 85^c^ ± 1 |

Values with different letters (^a-e^) in the same row are significantly different at p<0.05.

**Table S5**. Abundance of bacterial taxa in rats fed malt and BSG diets (Tipple malt, Cinnamon malt, Standard malt, Standard BSG, Mixture), barley extracts (Arabinoxylan extract, β-glucan extract) and the fibre-free control diet.

|  | Tipple  malt | Cinnamon  malt | Standard  malt | BSG | Mixture | AX  extract | β-glucan  extract | Control  (fibre-free) |
| --- | --- | --- | --- | --- | --- | --- | --- | --- |
| f *Coriobacteriaceae*; g unclassifed | 1.55 | 3.35 | 1.19 | 0.88 | 1.62 | 1 | 8 | 13 |
| g *Adlercreutzia* | 0.22 | 0.13 | 0.25 | 0.14 | 0.7 | 0.9 | 0.1 | 0.4 |
| o *Bacteroidales*; f, g unclassified | 0.31 | 0.02 | 0.33 | 0.22 | 0.2 | 0.0 | 0.0 | 0.0 |
| g *Bacteroides* | 6.17 | 2.18 | 6.54 | 4.81 | 3.6 | 16 | 6 | 2 |
| g *Parabacteroides* | 2.00 | 1.62 | 1.91 | 1.56 | 1.9 | 0.8 | 1.4 | 2.3 |
| g *Prevotella* | 0.26 | 0.18 | 0.60 | 0.37 | 0.9 | 2.2 | 0.1 | 0.0 |
| f *Rikenellaceae*; g unclassified | 3.34 | 1.50 | 5.89 | 4.26 | 3.9 | 1.0 | 0.5 | 2.6 |
| f *S24*-7; g unclassified | 5.84 | 3.27 | 8.59 | 5.27 | 5.8 | 3.5 | 3.7 | 2.0 |
| g *Odoribacter* | 0.08 | 0.02 | 0.21 | 0.15 | 0.1 | 0.0 | 0.0 | 0.0 |
| o *YS2*; f, g unclassified | 0.03 | 0.02 | 0.05 | 0.10 | 0.1 | 0.3 | 0.0 | 0.1 |
| f *Enterococcaceae*; g unclassified | 0.01 | 0.01 | 0.01 | 0.01 | 0.0 | 0.02 | 0.01 | 0.2 |
| g *Lactobacillus* | 26.17 | 36.68 | 8.42 | 26.76 | 28.5 | 1 | 19 | 4 |
| g *Turicibacter* | 0.06 | 0.05 | 0.24 | 0.08 | 0.0 | 0.8 | 0.3 | 1.0 |
| o *Clostridiales*; f, g unclassified | 19.91 | 15.52 | 20.10 | 29.74 | 26.2 | 20 | 8 | 13 |
| f *Clostridiaceae*; g unclassified | 0.09 | 0.07 | 0.33 | 0.11 | 0.2 | 0.2 | 0.1 | 0.0 |
| g *Clostridium* | 1.36 | 1.27 | 0.48 | 0.36 | 0.6 | 1 | 1 | 5 |
| f *Dehalobacterriaceae* | 0.06 | 0.10 | 0.09 | 0.01 | 0.0 | 0.1 | 0.0 | 0.1 |
| g *Dehalobacterium* | 0.09 | 0.01 | 0.08 | 0.04 | 0.0 | 0.0 | 0.0 | 0.1 |
| f *Lachnospiraceae*; g unclassified | 7.06 | 5.32 | 7.11 | 4.53 | 5.2 | 12 | 5 | 8 |
| g *Blautia* | 0.92 | 1.96 | 1.26 | 0.01 | 0.3 | 2 | 11 | 6 |
| g *Coprococcus* | 0.44 | 0.71 | 0.81 | 0.95 | 1.2 | 13 | 0 | 0 |
| g *Dorea* | 0.36 | 0.60 | 0.38 | 0.29 | 0.3 | 1 | 6 | 13 |
| g *Ruminococcus* | 4.04 | 3.13 | 5.38 | 6.44 | 5.5 | 0.3 | 0.3 | 3.8 |
| g *rc4-4* | 0.12 | 0.13 | 0.24 | 0.05 | 0.1 | 0.1 | 0.0 | 0.3 |
| f *Peptostreptococcaceae*; g unclassified | 0.01 | 0.01 | 0.02 | 0.01 | 0.0 | 0.02 | 0.01 | 0.01 |
| f *Ruminococcaceae*; g unclassified | 4.60 | 4.01 | 6.53 | 2.82 | 4.5 | 6 | 15 | 3 |
| g *Oscillospira* | 2.56 | 1.14 | 3.06 | 4.25 | 3.6 | 1.8 | 0.7 | 1.2 |
| g *Ruminococcus* (f *Ruminococcaceae*) | 0.73 | 0.43 | 0.96 | 0.38 | 0.7 | 0.6 | 0.5 | 0.1 |
| f *Mogibacteriaceae*; g unclassified | 0.30 | 0.27 | 0.56 | 0.16 | 0.6 | 0.1 | 0.1 | 0.4 |
| f *Halanaerobiaceae*; g unclassified | 1.55 | 1.01 | 2.68 | 1.43 | 1.1 | 3.7 | 1.1 | 1.2 |
| f *Erysipelotrichaceae*; g unclassified | 0.02 | 0.00 | 0.00 | 0.01 | 0.1 | 0.0 | 0.0 | 0.0 |
| g *Allobaculum* | 8.81 | 14.38 | 14.72 | 3.40 | 2.1 | 3 | 12 | 15 |
| g *Coprobacillus* | 0.03 | 0.10 | 0.08 | 0.00 | 0.0 | 0.0 | 0.0 | 0.0 |
| g *Eubacterium* | 0.00 | 0.02 | 0.01 | 0.00 | 0.0 | 0.0 | 0.1 | 0.1 |
| g *Bilophila* | 0.01 | 0.00 | 0.01 | 0.02 | 0.0 | 0.0 | 0.0 | 0.0 |
| f *Enterobacteriaceae*; g unclassified | 0.00 | 0.00 | 0.00 | 0.04 | 0.0 | 0.0 | 0.0 | 0.1 |
| o *RF39*; f, g unclassified | 0.13 | 0.12 | 0.20 | 0.09 | 0.1 | 0.2 | 0.0 | 0.2 |
| g *Akkermansia* | 0.76 | 0.66 | 0.68 | 0.26 | 0.2 | 7 | 1 | 0 |

**Table S6**. Correlation of dietary fibre with the abundance of some microbiota species in rats fed the malt and BSG diets (Tipple malt, Cinnamon malt, Standard malt, Standard BSG, Mixture).

|  | % Soluble  fibre | Total  arabinoxylan | % Soluble  arabinoxylan | β-glucan | β-glucan  Mw |
| --- | --- | --- | --- | --- | --- |
| g *Bacteroides* ¤ | NC | NC | NC | NC | NC |
| g *Prevotella* | NC | NC | NC | NC | NC |
| f *Rikenellaceae*; g unclassified ¤ * | -- | ++ | - | -- | - |
| f *S24*-7 g; unclassified ¤ * | - | + | NC | - | NC |
| g *Odoribacter ** | - | ++ | - | - | - |
| g *Lactobacillus* ¤ * | + | - | NC | + | NC |
| o *Clostridiales*; f, g unclassified ¤ * | -- | + | -- | - | - |
| g C*lostridium ** | + | - | + | ++ | + |
| g *Blautia ** | ++ | - | ++ | + | + |
| g *Coprococcus ** | NC | NC | NC | NC | NC |
| g *Dorea* | NC | NC | NC | NC | NC |
| g *Ruminococcus* (f.*Lachnospiraceae*) ¤ * | -- | ++ | -- | -- | -- |
| g *rc4-4 ** | NC | NC | NC | NC | NC |
| f *Ruminococcaceae*; g unclassified ¤ | NC | NC | NC | NC | NC |
| g *Oscillospira ** | -- | + | -- | - | - |
| g *Allobaculum* ¤ | + | NC | + | NC | NC |
| g *Coprobacillus ** | + | NC | + | NC | NC |
| f *Enterobacteriaceae*; g unclassified *** | - | NC | - | - | - |
| g. *Akkermansia* | + | NC | + | + | + |

a) * taxa significantly different between diets, (p<0.05, ANOVA); ¤ abundance >5%; NC – no correlation.

b) Plus/minus symbol indicates the positive/negative correlation between SCFA and bacteria. In absolute values: + or –, 0.25< r <0.50; ++ or – –, 0.50< r <0.75; +++ or – – –, 0.75< r <1.00.


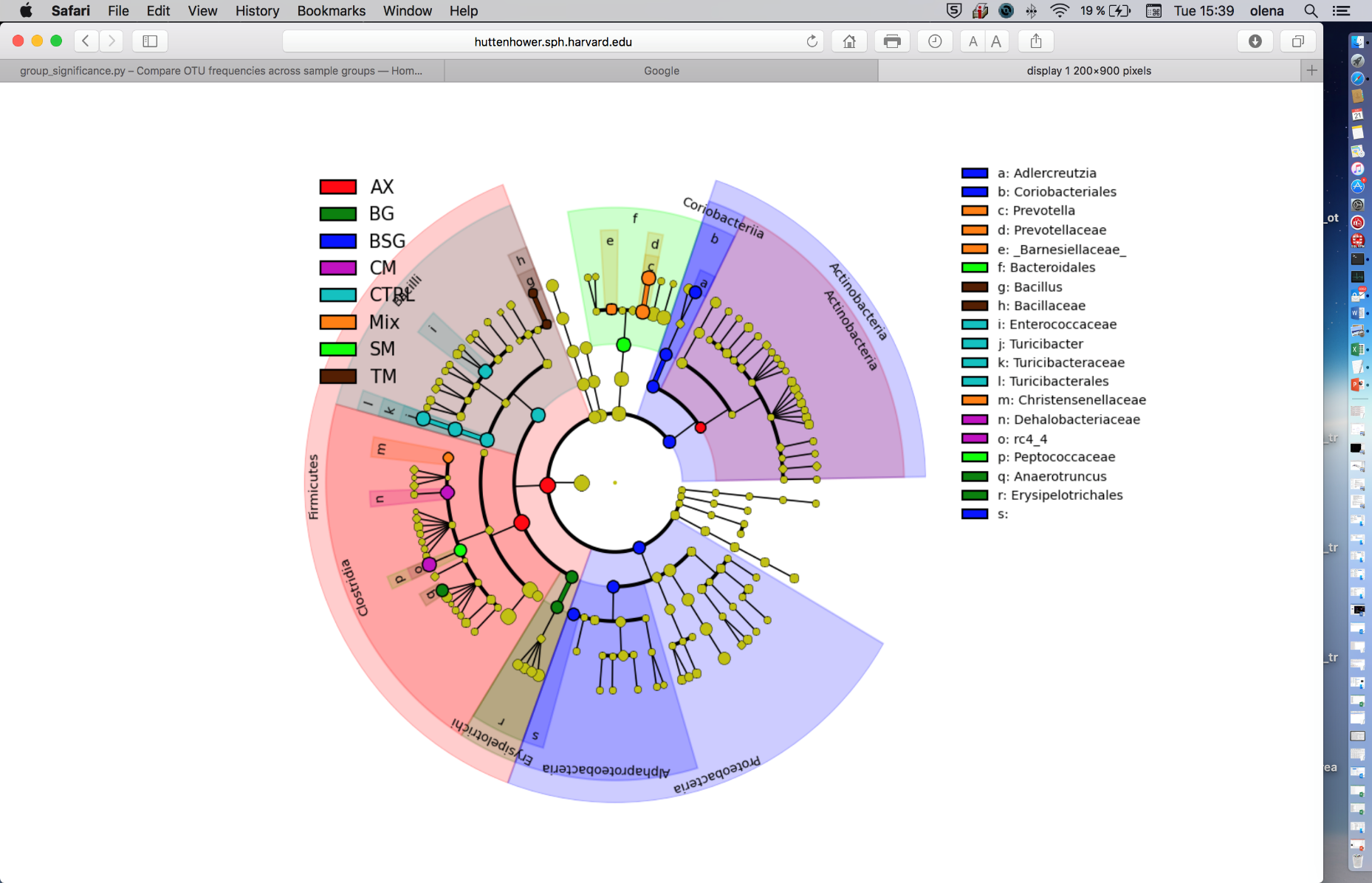

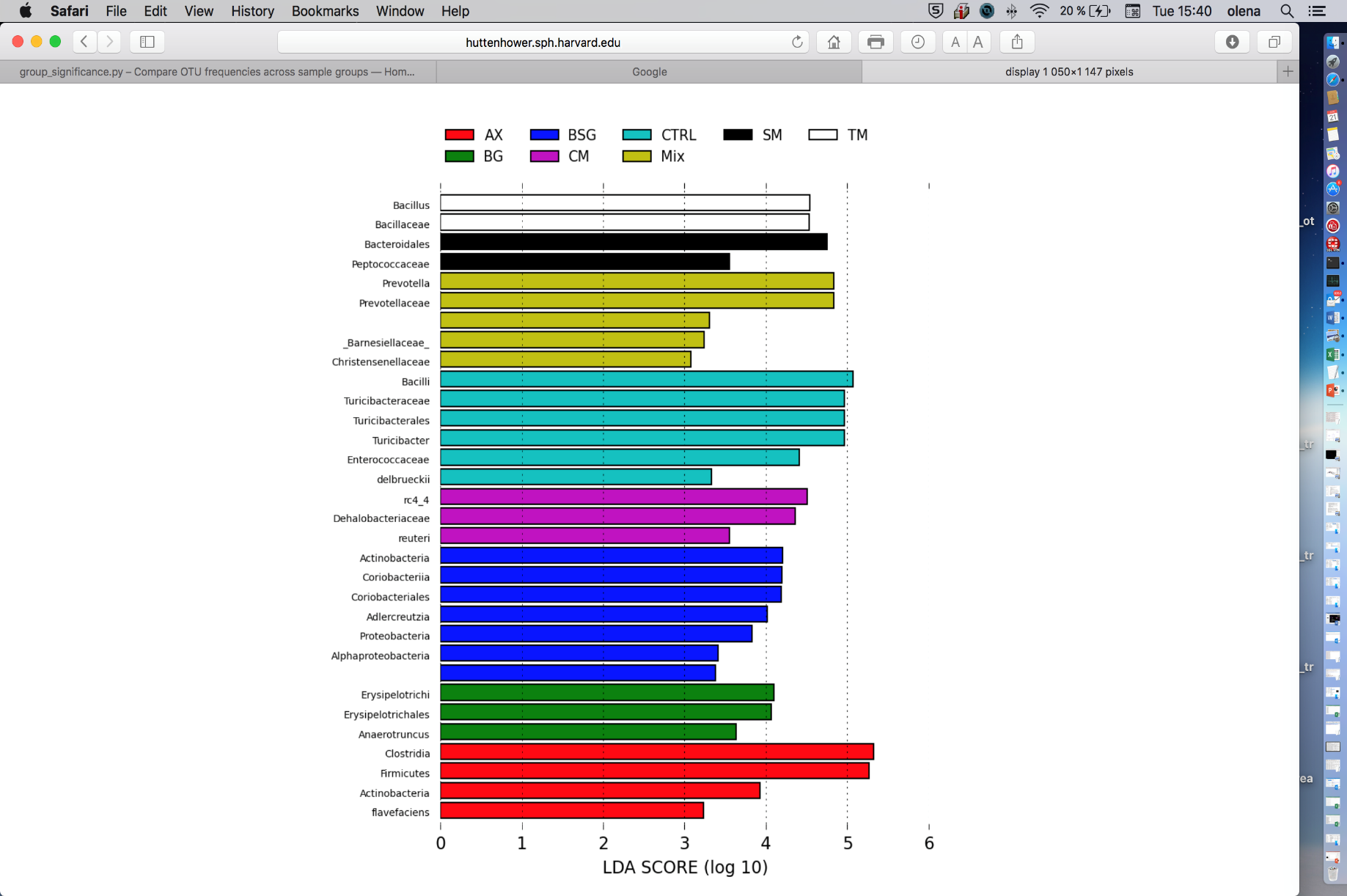


**Figure S1**

Linear discriminant analysis (LDA) score plot (left), indicates the relative abundance in the corresponding group compared with all the other groups. Taxonomy cladogram (right), colours refer to the taxa in the different diet groups, and the circle size is based on relative abundance.


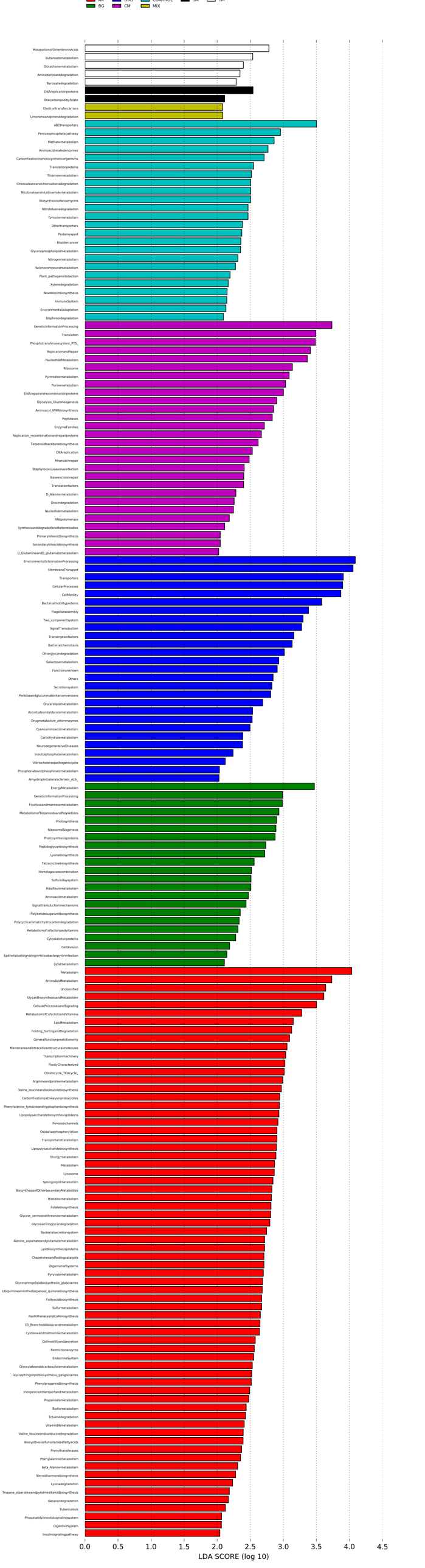

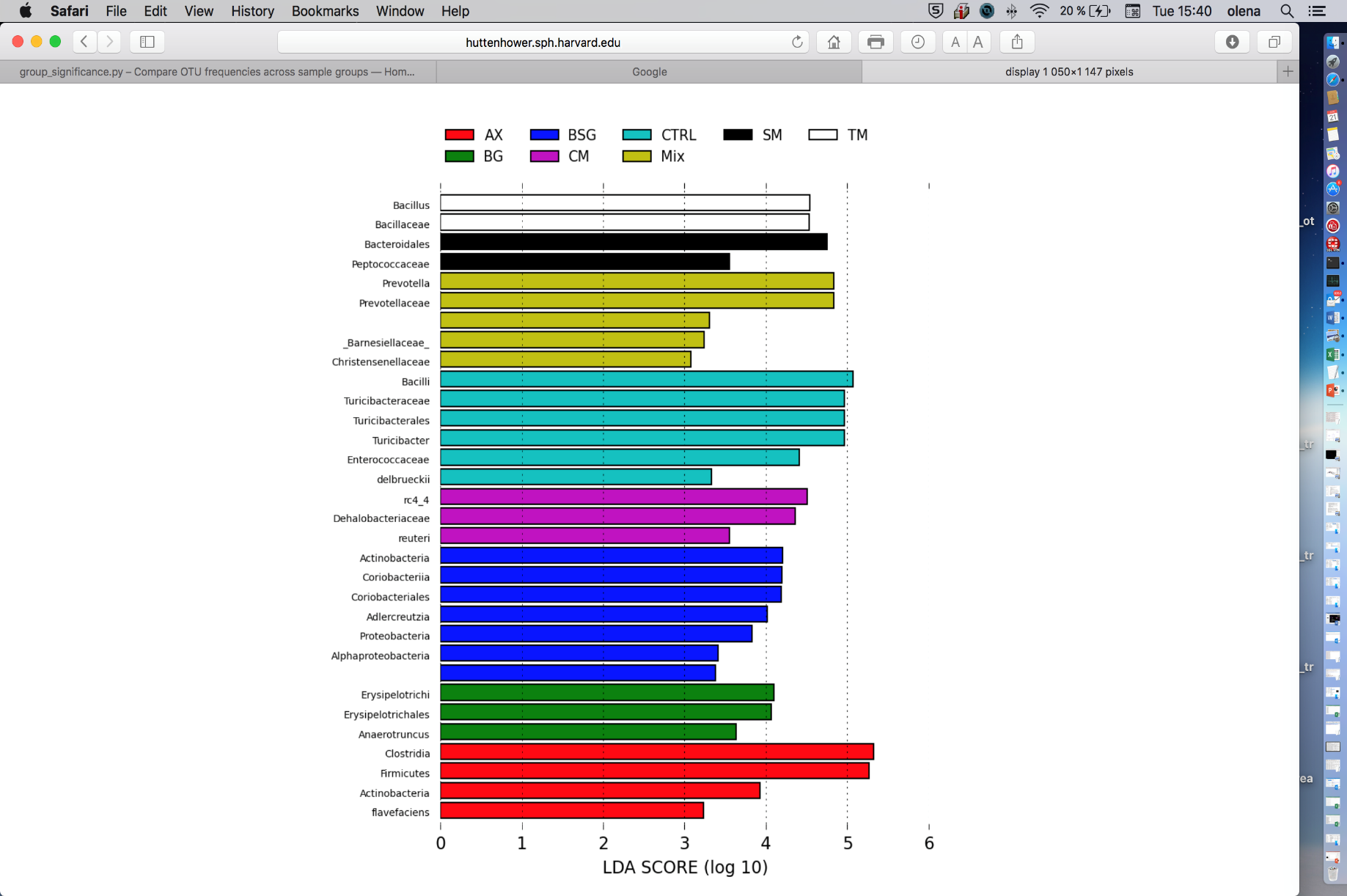


**Figure S2a**

PICRUSt analysis of 16S rRNA gene sequencing data. Functional microbial genes shown were enriched in the respective groups and had an LDA score higher than 2, for Tipple malt, Cinnamon malt, Standard malt, BSG and Mixture groups.


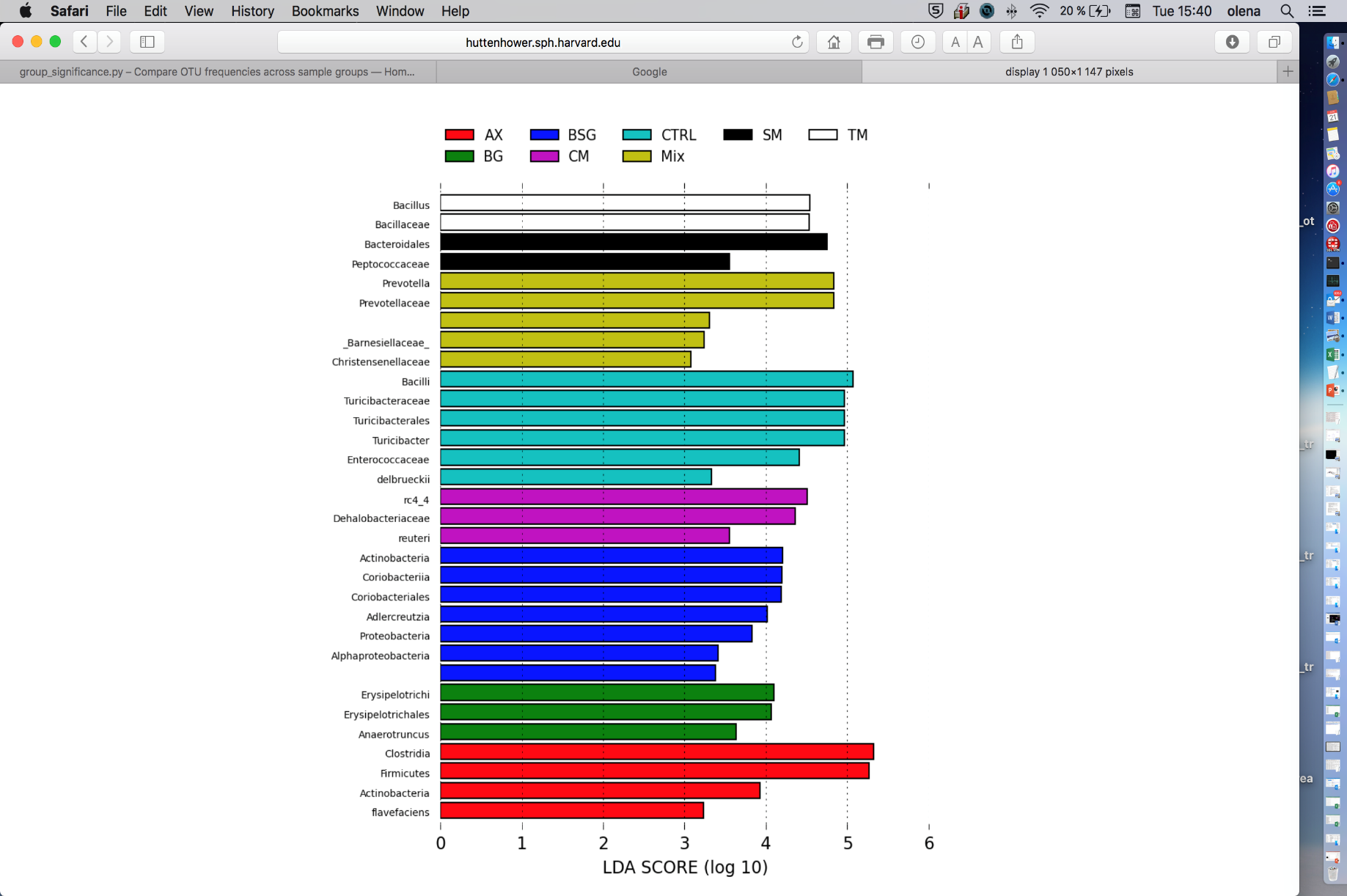

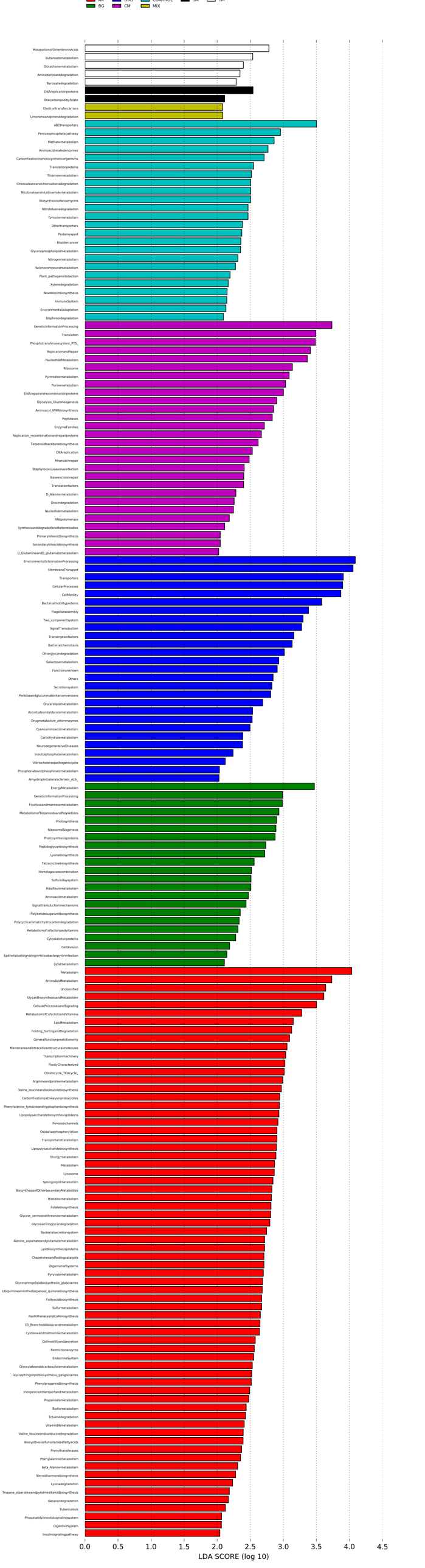


**Figure S2b**

PICRUSt analysis of 16S rRNA gene sequencing data. Functional microbial genes shown were enriched in the respective groups and had an LDA score higher than 2, for BG extract and AX extract groups.
