# Supplementary material for: Barley Products of Different Fiber Composition Selectively Change Microbiota Composition in Rats
Source: Mol Nutr Food Res. 2018 Aug 12;62(19):1701023. doi: 10.1002/mnfr.201701023 (PMC6175208; doi:10.1002/mnfr.201701023)
Supplement: Supplementary file 3 — Supporting Information [file MNFR-62-na-s003.docx]

**Biom summary tables**

***-- Before filtering –***

Num samples: 56

Num observations: 3235

Total count: 27858485

Table density (fraction of non-zero values): 0.300

Counts/sample summary:

Min: 212070.0

Max: 840486.0

Median: 514010.500

Mean: 497472.946

Std. dev.: 129344.981

Sample Metadata Categories: None provided

Observation Metadata Categories: taxonomy

Counts/sample detail:

3D: 212070.0

8D: 280446.0

5D: 290614.0

3B: 315083.0

4C: 325114.0

6E: 327475.0

6C: 329012.0

6F: 343297.0

7B: 354584.0

6A: 358158.0

1C: 373674.0

7G: 379469.0

1A: 385386.0

8C: 387916.0

4D: 394343.0

6G: 396944.0

5G: 401076.0

3A: 416635.0

5A: 421510.0

3G: 423720.0

6B: 423983.0

1B: 430948.0

1F: 469548.0

4E: 484807.0

4A: 493008.0

2D: 507138.0

5E: 508618.0

6D: 511279.0

3F: 516742.0

3C: 524905.0

8G: 526378.0

2A: 527460.0

1E: 529434.0

2F: 531697.0

2E: 537144.0

4F: 537476.0

4G: 553527.0

7F: 565852.0

5C: 567685.0

1G: 574856.0

1D: 577101.0

5F: 585094.0

3E: 597465.0

8E: 608008.0

8A: 618357.0

8B: 619250.0

8F: 620727.0

2B: 638559.0

5B: 639144.0

7A: 651954.0

2G: 657124.0

7C: 666241.0

4B: 674010.0

2C: 685109.0

7D: 740845.0

7E: 840486.0

***-- After filtering --***

Num samples: 56

Num observations: 431

Total count: 27294547

Table density (fraction of non-zero values): 0.800

Counts/sample summary:

Min: 206546.0

Max: 828678.0

Median: 503802.500

Mean: 487402.625

Std. dev.: 128532.430

Sample Metadata Categories: None provided

Observation Metadata Categories: taxonomy

Counts/sample detail:

3D: 206546.0

8D: 271115.0

5D: 282428.0

3B: 304792.0

4C: 311597.0

6C: 319411.0

6E: 319607.0

6F: 337828.0

7B: 347164.0

6A: 352614.0

1C: 363144.0

1A: 370888.0

7G: 372396.0

8C: 379914.0

4D: 385579.0

6G: 388099.0

5G: 389045.0

3A: 408024.0

3G: 409192.0

5A: 409694.0

6B: 412996.0

1B: 420144.0

1F: 461925.0

4E: 474825.0

4A: 480918.0

2D: 500226.0

5E: 501946.0

6D: 502707.0

3F: 504898.0

8G: 514102.0

3C: 515808.0

1E: 520783.0

2F: 521078.0

2A: 521995.0

2E: 527171.0

4F: 528148.0

4G: 541790.0

1D: 552167.0

5C: 553323.0

7F: 558813.0

1G: 563458.0

5F: 571923.0

3E: 589277.0

8E: 598659.0

8A: 610401.0

8F: 611207.0

8B: 614538.0

5B: 629648.0

2B: 633019.0

7A: 635767.0

7C: 647302.0

2G: 648105.0

4B: 663808.0

2C: 679040.0

7D: 724877.0

7E: 828678.0
